# Transactions of the American Medical Association

**Published:** 1854-03

**Authors:** 


*Transactions of the American Medical Association.*

WE see by our exchanges that the sixth volume of the Transactions of the American Medical Association is published. Those wishing it should send in their orders immediately. We know nothing of its contents excepting what we have learned from the notices of our exchanges. J.

